# Thrombectomy of a Complex Deep Vein Thrombosis: A Case Report

**DOI:** 10.7759/cureus.33413

**Published:** 2023-01-05

**Authors:** Talha Shabbir, Matthew Wade, Krishna Das, Rahul Nayyar

**Affiliations:** 1 Research, California University of Science and Medicine, Colton, USA; 2 Radiology, Victor Valley Global Medical Center (KPC Health), Victorville, USA

**Keywords:** deep vein thrombosis, ivc filter, thrombus, interventional radiology, wells dvt score, endovascular thrombectomy, oral anticoagulation, deep vein thrombosis (dvt)

## Abstract

A deep vein thrombosis (DVT) is a multifactorial condition characterized by a thrombus or blood clot developing in the deep veins of the upper or lower extremities. The presentation of DVT is characterized by unilateral peripheral edema and signs of inflammation in the affected extremity. The treatment of DVT is complex and case-dependent; however, most individuals are managed with oral anticoagulation therapy, while complex cases can be treated with advanced interventions. This report discusses a singular case of an atypical or complex DVT in a middle-aged woman with prior venous thromboembolic events undergoing a thrombectomy using an Inari FlowTriever system.

## Introduction

A deep vein thrombosis (DVT) is a multifactorial condition characterized by a thrombus or blood clot developing in the deep veins of the upper or lower extremities [1,2]. While age, sex, obesity, smoking, and various other factors play a role in developing thrombosis, the most prominent risk factors are venous stasis, endovascular damage, and hypercoagulability, or thrombophilia [2]. Collectively, these factors are referred to as Virchow’s Triad [1,2].

In hemostasis, endothelial wall damage and underlying inflammatory processes expose subendothelial collagen, thus activating clotting factors that lead to downstream platelet aggregation and thrombus formation [2,3]. While a thrombus can develop spontaneously, underlying genetic or acquired thrombophilias such as coagulation cascade factor disorders, platelet disorders, malignancies, oral contraceptive medications, or infections can drastically increase the susceptibility to thrombosis [2-4]. Increased venous stasis stemming from prolonged immobilization or vascular pathologies can further enhance the production of DVTs [1,4,5]. 

The presentation of DVT is often accompanied by unilateral peripheral edema and signs of inflammation, such as warmth, swelling, and pain in the affected extremity [1,2,5,6]. Severe complications of DVT arise when ruptured pieces of the thrombus, referred to as emboli, travel within the bloodstream and become lodged in small vessels of various organ systems, such as the lungs [1,2].

The treatment of DVT is complex and case-dependent; however, most individuals are managed with oral anticoagulation therapy [1,2,5,6]. Complex cases can be treated with advanced interventions such as catheter-directed thrombolysis and thrombectomy [7].

This report discusses a singular case of an atypical or complex DVT in a middle-aged woman with prior venous thromboembolic events who underwent a thrombectomy using an Inari FlowTriever system.

## Case presentation

A morbidly obese 43-year-old woman with a significant past medical history of left lower extremity deep vein thrombosis (DVT), pulmonary embolism (PE), cerebrovascular accident (CVA), and placement of an inferior vena cava (IVC) filter presented to the emergency department with right lower extremity swelling and tenderness over two days. She was previously on Coumadin and Xarelto for six months due to her history of DVT and PE; however, she was not on anticoagulant therapy upon presentation. During her visit, a review of her symptoms was positive only for swelling and joint pain. The vital signs obtained were stable, and the physical exam revealed an oriented woman with clear lungs, normal heart sounds, a soft abdomen, and a swollen right leg. Laboratory findings indicated a white blood count of 8.8 × 109/L, hemoglobin of 11.9 g/dL, hematocrit of 33.2%, platelets of 344 × 109/L, and potassium of 3.1 mEq/L. Additional findings noted were a prothrombin time (PT) of 13.1 seconds, a partial thromboplastin time (PTT) of 38 seconds, and an international normalized ratio (INR) of 1.16. Based on her clinical presentation and significant medical history, an initial Well’s score was determined to be greater than two, thus placing her in the high-risk category for DVT. A Doppler ultrasound evaluation of the deep venous system of the right lower extremity from the common femoral vein to the popliteal vein was performed, including color and spectral waveform analysis. Imaging revealed intraluminal thrombus in the common femoral vein, superficial femoral vein, deep femoral vein, and popliteal vein. Due to extensive thrombus burden, extremity pain with swelling, and a prior history of thrombosis, a plan was made for a right lower extremity venogram and thrombectomy. The patient was admitted to the hospital and started on Lovenox for DVT prophylaxis, Dilaudid for pain control, and Zofran for nausea. A follow-up ventilation-perfusion (VQ) scan ruled out a PE. Lastly, the patient was placed on the nothing-by-mouth (NPO) protocol with intravenous (IV) fluids in preparation for intervention the following day.

**Figure 1:**
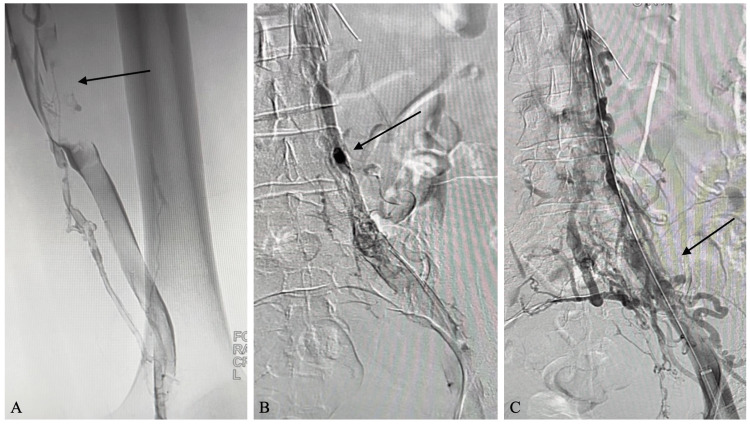
Pre-thrombectomy venogram (A) The initial venogram demonstrates significant thrombus in the iliac, femoral, and popliteal veins; (B) The pre-thrombectomy venogram shows extensive clot formation and collateral veins; (C) A large volume clot is present with collaterals in the venous system and an IVC filter already in place. IVC: inferior vena cava

Before the procedure, the patient was sedated, placed prone on the angiographic table, and prepped sterilely. An ultrasound-guided percutaneous puncture of the right popliteal vein was performed using the micropuncture Seldinger technique. Next, an exchange was made to the 0.035-inch system, and a 5-French Kumpe catheter and Glidewire were inserted and navigated into the inferior vena cava (IVC). The venogram showed a large thrombus involving the iliac, femoral, and popliteal veins and extensive collateral formation (Figure 1).

**Figure 2:**
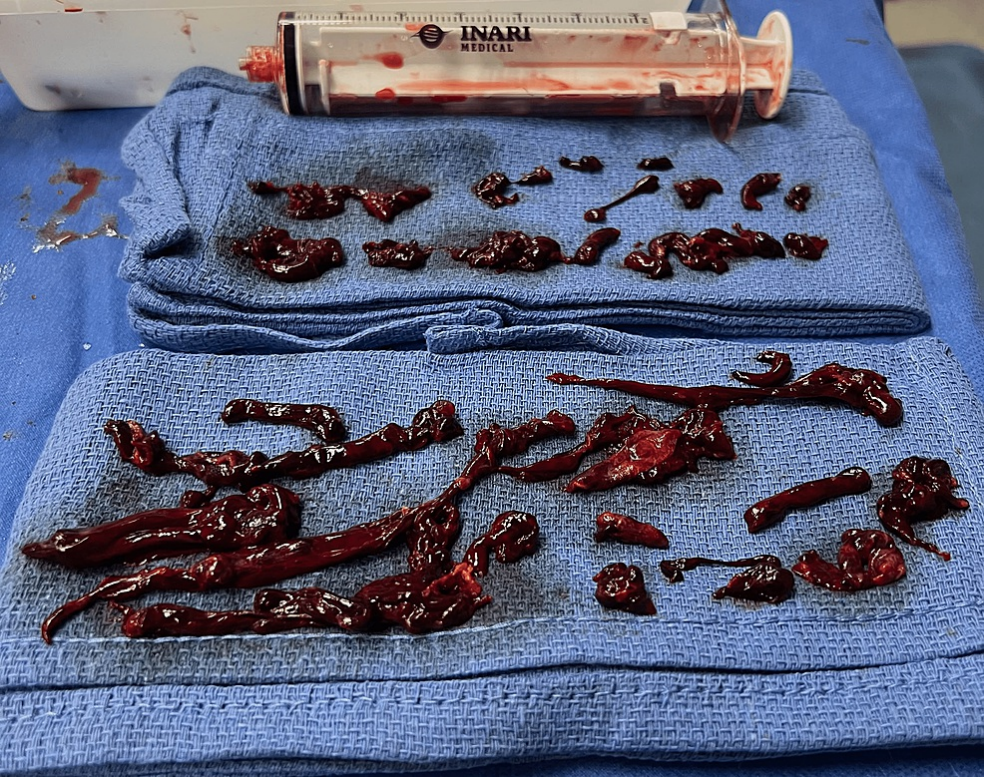
Acute, subacute, and chronic thrombus were retrieved over an 80-minute period using the Inari FlowTriever system

At this time, a decision was made to proceed with a thrombectomy. Serial dilations were performed, and a 22 French Gore DrySeal sheath was inserted into the popliteal vein over an Amplatz guidewire. Next, the Inari thrombectomy devices (Inari Medical, Irvine, California), including Triever20 and Triever16, were placed over the wire, and a total of 15 "Whoosh" aspirations, over 80 minutes, were used to retrieve an extraordinary amount of acute, subacute, and chronic thrombus (Figure 2).

**Figure 3:**
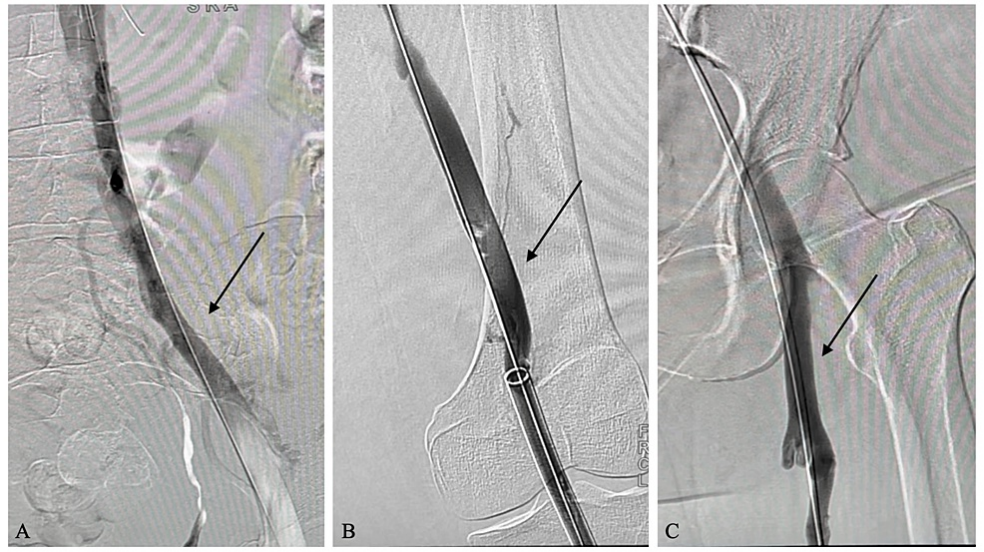
Post-thrombectomy venogram (A) A post-thrombectomy venogram showing brisk flow in the venous system and resolution of collaterals; (B) brisk venous flow in the superficial femoral vein; (C) brisk flow in the common femoral vein. No clot is seen post-treatment.

A total of 750 ml of blood was aspirated; however, the FlowSaver Blood Return System (Inari Medical, Irvine, California) was utilized to minimize blood loss to 100 ml. The patient received multiple boluses of 3000 units of heparin throughout the procedure, with no thrombolytics administered. A post-thrombectomy venogram showed 85% narrowing at the right iliac vein and IVC junction. As such, venoplasty was performed on the vessel using a 14-millimeter balloon, resolving the stenosis. Additional venograms demonstrated moderate thrombus just below the IVC filter; thus, the filter was not removed. A brisk, patent flow was demonstrated throughout the right venous system on the final venogram (Figure 3), and the procedure was subsequently concluded.

The patient did not require a stay in the intensive care unit (ICU) following the procedure. Instead, she stayed in the inpatient unit for six days from admission to discharge, with continuous improvements in her symptoms after the thrombectomy. She remained stable and was discharged with recommendations for continued anticoagulation and a repeat venogram in three months. The patient’s anticoagulation therapy included a 10-milligram dose of Eliquis to be taken twice a day and reduced to 5 mg after 10 days.

## Discussion

The patient presented with classic signs of deep vein thrombosis, including right lower extremity edema and tenderness. Once thrombosis was suspected, the workup began with a pre-test probability formulated using Wells Criteria (Table 1).

**Table 1:** Pre-test probability (Wells score) A pre-test probability can be tabulated using Wells criteria [2,6]. A Wells score of less than two and a negative D-dimer test can be used to rule out a DVT [5]. Conversely, a score greater than two, or less than two with a positive D-dimer, indicates a venous ultrasound examination of the affected extremity to assess the presence of a thrombus [1,2,4,6]. DVT: deep vein thrombosis

Features	Points
Active cancer	+1
Paralysis, paresis, or recent plaster immobilization of the lower extremities	+1
Recently bedridden for >3 days or major surgery within 12 weeks	+1
Localized tenderness along the distribution of the deep veins	+1
Entire leg swollen	+1
Calf swelling ≥ 3 cm compared to asymptomatic leg	+1
Pitting edema limited to the symptomatic leg	+1
Collateral superficial (non-varicose) veins present	+1
Previous DVT	+1
Alternative diagnosis; likely as or more likely than DVT	-2

Typically, patients with a Wells score of less than two are further worked up with a D-dimer test [1,2,4,6]. Although a D-dimer test is highly sensitive to thromboembolic events, the specificity is rather poor [4,5]. Therefore, a positive D-dimer test (≥500 ng/mL) does not necessarily indicate the presence of a DVT. However, after a Wells score of less than two, a negative D-dimer test can rule out a DVT [5]. Conversely, if the Wells score is greater than or less than two with a positive D-dimer, a venous ultrasound examination of the affected extremity is indicated to assess the presence of a thrombus [1,2,4,6]. In this patient’s case, a history of prior thromboembolic events and right leg swelling with tenderness placed her in the high-risk category with a Wells score greater than two. As such, a venous ultrasound was ordered, confirming thromboses in multiple vessels throughout the right lower extremity. 

Once a thrombus is found, treatment typically involves oral anticoagulation therapy. Examples of therapeutics include direct oral anticoagulants (DOACs), vitamin K antagonists (VKAs), and low molecular weight heparin (LMWH) [1,2,5,6]. In limb-threatening ischemia or complicated DVTs, advanced interventions can be utilized, including catheter-directed thrombolysis, thrombectomy, and IVC filters [1,2]. In this case, the patient required a thrombectomy due to her history of thromboembolic events and a complex DVT exemplified by the extensive thrombotic disease in her leg.

Catheter-directed interventions include percutaneous mechanical thrombectomy devices, which can break down and remove thrombosis, and catheter-directed thrombolysis devices, which can administer thrombolytics or pharmaceutical drugs directly into a thrombosed vein [8,9]. ClotTriever, AngioVac, and Indigo Aspirational Systems are among the mechanical thrombectomy devices [8,9]. Devices such as the Cleaner Rotational, AngioJet, and JETi Hydrodynamic Thrombectomy Systems are used for mechanical thrombectomy and catheter-directed thrombolysis [8,9]. Lastly, the EkoSonic Endovascular System can achieve thrombolysis; however, in conjunction with ultrasound imaging, it can be used for mechanical thrombectomy as well [8].

In this patient, the thrombectomy procedure gained access through the right popliteal vein via a Gore DrySeal sheath. The Inari FlowTriever system was then delivered into the thrombus via wire. Typically, these systems are reserved for retrieval of pulmonary embolisms after the Favipiravir +/- Lopinavir: A RCT of Early Antivirals (FLARE) trial demonstrated safe and efficacious intervention [7]. However, these systems can rarely be utilized for other large vessels and have been effective in complex DVT interventions [10,11]. Specifically, in this patient, the FlowTriever system retrieved a significant amount of thrombus without complications. Afterward, the patient did not require a stay in the ICU, showed symptomatic improvement, and was discharged a few days later with pharmacologic management.

## Conclusions

Deep vein thrombosis is a rare condition with the highest prevalence in elderly and obese individuals. This case report elaborates on a patient with an atypical DVT who underwent a thrombectomy after venous ultrasound exemplified extensive thrombotic disease in the right lower extremity. In addition to the extensive thrombus burden, the presence of an IVC filter and IVC thrombus further enhanced the complexity of the case. During the procedure, the Inari FlowTriever system, though typically indicated for pulmonary embolism interventions, was utilized and successfully retrieved a significant amount of thrombus. A post-procedural venogram indicated improvements in flow throughout the right lower extremity venous system, and the patient showed symptomatic improvement without reporting complications.
